# Prostate- and metastases-directed radiotherapy in de novo low-volume metastatic prostate cancer

**DOI:** 10.1016/j.ctro.2025.101072

**Published:** 2025-11-14

**Authors:** Jan-Hendrik Bolten, Fabian Weykamp, Christoph Grott, David Neugebauer, Lars Wessel, Felix H. Englert, Justus Valentini, Magdalena Goertz, Stephanie Zschaebitz, Johannes Huber, Erik Winter, Juergen Debus, Jakob Liermann

**Affiliations:** a Departement of Radiation Oncology, University Hospital Heidelberg, Germany; b Clinical Cooperation Unit Radiation Oncology, German Cancer Research Center (DKFZ), Heidelberg, Germany; c National Center for Tumor Diseases (NCT), Departement of Radiation Oncology, Heidelberg, Germany; d Heidelberg Institute of Radiation Oncology (HIRO), University Hospital Heidelberg, Germany; e Junior Clinical Cooperation Unit, Multiparametric Methods for Early Detection of Prostate Cancer, German Cancer Research Center (DKFZ), Heidelberg, Germany; f Department of Urology, University Hospital Heidelberg, Germany; g National Center for Tumor Diseases (NCT), Departement of Medical Oncology, Heidelberg, Germany; h Departement of Nuclear Medicine, University Hospital Heidelberg, Germany

**Keywords:** Excellent tolerability of ablative radiotherapy of all PSMA-PET positive tumor manifestations in low-volume disease of oligometastatic prostate cancer, Importance of PSMA-PET-Staging in oligometastatic prostate cancer, Further stratification in low volume disease of oligometastatic prostate cancer is needed

## Abstract

**Background:**

The role of radiotherapy in de novo low-volume metastatic prostate cancer (LVmPC) is constantly evolving and still offers considerable potential for further optimization. Rising interest in this topic demands further investigations in this specific patient cohort. We report on clinical outcomes and toxicity of combined prostate-directed (PDRT) and metastasis-directed radiotherapy (MDRT) with ablative dose concepts in patients with de novo LVmPC.

**Methods:**

We retrospectively analyzed 21 patients with LVmPC treated with PDRT plus MDRT between 2018 and 2025 in addition to systemic treatment. Staging was performed with PSMA-PET/CT imaging (n = 19/21). Radiotherapy included high-dose external beam PDRT and MDRT to all detectable bone and nodal metastases. Toxicity was assessed using CTCAE v5.0, IPSS, and EPIC-26 questionnaires. Oncologic outcomes included biochemical recurrence-free survival, PSA response, and time to next-line systemic therapy.

**Results:**

Additional radiotherapy of all tumor sites in LVmPC was well tolerated; no grade ≥ III gastrointestinal or genitourinary toxicity occurred. The most common adverse events were associated with hormonal therapy. Quality of life remained stable or improved in urinary and bowel domains. Undetectable PSA was achieved in 67 % of patients after radiotherapy in addition to systemic therapy. 14 % of the patients developed biochemical recurrence within 40 months after radiotherapy completion.

**Conclusion:**

Combined PDRT and MDRT in addition to standard of care in patients with de novo LVmPC is feasible, well tolerated, and associated with promising biochemical control. Further prospective studies with larger patient cohorts integrating PSMA-PET/CT and other risk stratification factors are warranted to better define which patient subgroups benefit most from additional radiotherapy.

## Highlights

• Excellent tolerability of combined prostate- and metastases-directed radiotherapy.
• Pre-treatment PSMA-PET suggests therapeutic advantage.
• Potential curative therapy approach in de novo low-volume prostate cancer.

## Background

De novo metastatic hormone-sensitive prostate cancer (mHSPC) is rare with 5–10 % of all prostate cancer (PC) diagnoses and associated with worse prognosis than localized and metachronous metastatic PC. [[1], [2], [3]] The incidence of mHSPC has increased with the routine use of PSMA-PET/CT imaging in high-risk PC staging and is expected to continue rising. [4] James et al. project an increase of over 100 % for PC in general within the next two decades. [5] The precise role and optimal use of radiotherapy in oligometastatic PC remains uncertain and needs further investigation.

In patients with localized PC, surgery and radiotherapy (RT) offer high rates of disease-free survival (DFS) and a five-year overall survival (OS) exceeding 90 %, depending on the individual risk profile. By contrast, in the metastatic setting, systemic therapy remains the standard of care with a median OS from 3.1 to 5.1 years depending on risk constellation. [[6], [7], [8], [9]] A further risk stratification in metastatic prostate cancer (mPC) patients is highly-recommended for therapy selection. Next to basic parameters like TNM classification, Gleason-score and prostate specific antigen value (PSA) a distinction between high- and low-volume metastatic PC (LVmPC) should be made, as well as metachronous or synchronous (de novo) metastasis. [10,11] These factors contribute to the differentiation and classification of distinct risk groups, which are crucial for both, therapeutic decision-making and prognosis.

De novo mPC seems more aggressive with even worse survival and shorter time to castration resistance compared to metachronous mPC. [12] Key limitations of systemic treatments in mPC include the need for continuous administration, time-limited efficacy, costs and a considerable burden of side effects. Patients with LVmPC are thought to have a more favorable prognosis compared to those with widespread metastatic disease. The definition of “low-volume” disease includes PC patients with less than four bone metastases limited to the vertebral bodies and pelvis and no visceral metastases based on computed tomography (CT) imaging as it was introduced in the CHAARTED trial. [11] Due to the excellent tolerability of both prostate-directed radiotherapy (PDRT) and stereotactic body radiotherapy (SBRT) for bone metastases, there is growing interest in combining palliatively intended systemic therapy with locally ablative RT approaches. [[13], [14], [15]].

Several studies have demonstrated potential benefits of additional RT in patients with mPC; however, it remains a matter of debate which patient subgroups derive benefit from specific RT strategies.

Several retrospective studies have suggested an OS benefit of PDRT in patients with mPC. [[16], [17], [18]] This finding was confirmed in the prospective STAMPEDE trial for patients with low-volume disease. [8] However, the recently published PEACE-1 trial did not confirm the significant OS benefit of PDRT in this setting but demonstrated a beneficial effect in local symptom control even for high-volume mPC. [9].

SBRT has demonstrated excellent local control of osseous metastases, particularly in the setting of oligorecurrence, and has been associated with improved progression-free survival (PFS) and prolonged androgen deprivation therapy (ADT)–free survival. [13,15,19].

The EXTEND trial already demonstrated a benefit in PFS of metastases-directed radiotherapy (MDRT) combined with intermittent ADT and androgen pathway inhibitors (ARPIs). Further ongoing trials (EORTC 2238 or Alliance A032101) evaluating the feasibility of intermittent ADT and ARPIs as possible new standard of care. [20,21] Given the various benefits and favorable tolerability of both PDRT and MDRT, it is increasingly being discussed whether the combination systemic treatment with a combined PDRT and MDRT should be investigated as a potentially curative approach in patients with LVmPC, especially considering therapeutic approaches using intermittent systemic therapy. However, data for combined PDRT and MDRT in PC patients are rare. [22].

In this study we retrospectively analyzed clinical outcomes of patients with de novo LVmPC who received a combined PDRT and MDRT with ablative dose regimens in addition to systemic therapy focused on feasibility, tolerability and efficiency.

## Methods

### Study design

We retrospectively analyzed all patients with LVmPC started a systemic therapy combined with PDRT and MDRT between 2018 and 2024 at our institution. All patients received a combined PDRT and MDRT (lymph node and bone metastases) of all radiographically definable tumor manifestations after a neoadjuvant systemic therapy, if applicable. The primary objective is to assess the tolerability and long-term side effects of RT to demonstrate its safety and the absence of negative impact on quality of life.

### Diagnosis of nodal and bone metastases

All patients received at least a conventional staging of abdominal and thoracic CT scans and bone scintigraphy. Additionally, 19 of 21 received a F18- or Ga68-prostate-specific membrane antigen positron emission tomography (PSMA-PET) as staging diagnostic. Lymph node metastases were diagnosed based on findings from PSMA-PET/CT and pelvic MRI. Bone metastases were identified using a bimodal approach, incorporating both metabolic and morphological criteria. Lesions were classified as bone metastases if they were PSMA-PET-positive or exhibited increased uptake on bone scintigraphy, with a corresponding morphological correlate on CT or MRI.

### Procedures

All patients were recommended to undergo standard of care systemic therapy. Upfront systemic therapy was not standardized but was administered at the discretion of the referring external urologists/oncologists. In our institutional practice, neoadjuvant ADT is typically administered for a minimum duration of two months and may be extended up to six months. Adjuvant systemic therapy was likewise determined by the treating physician; however, recommendations were provided by the institution’s internal interdisciplinary tumor board. At the time of RT planning, all imaging-detectable metastases were targeted with external beam RT. All patients diagnosed with N1 or M1a disease received whole pelvic RT with an integrated node directed dose-escalation (simultaneous integrated boost concept). Various ablative dose regimens were applied, depending on physician’s choice. RT of all lesions had to be completed within three months.

### Outcomes

The primary analyzed objects were treatment-related toxicity and patient-reported quality of life. Acute (adverse events during radiotherapy and within 3 months after RT completion) and late (adverse events occurred after more than 12 months after RT completion) toxicity was assessed using the Common Terminology Criteria for Adverse Events (CTCAE), version 5.0. [23] Adverse events were grouped by genitourinary, gastrointestinal, sexual (libido and erectile dysfunction), hormonal (hot flashes, breast tenderness, fatigue, weight loss) and bone damages. Treatment- and tumor-related symptom burden was assessed using the International Prostate Symptom Score (IPSS) and the Expanded Prostate Cancer Index Composite (EPIC-26) questionnaires collected before, within the first three months and more than 12 months after radiotherapy.

Further important objects of this study were biochemical recurrence-free survival (bRFS), defined according to the Phoenix criteria as a PSA increase of ≥2 ng/ml above the nadir; local control (LC), defined as the absence of radiographic progression at irradiated metastatic or primary tumor sites; and time to next-line systemic therapy, measured from the end of RT to the initiation of an additional subsequent systemic treatment or change of systemic treatment. [24] Another important endpoint is the PSA nadir within 6 to 12 months after initiation of systemic therapy. [[25], [26], [27]].

### Statistics and ethics

Descriptive statistics were used to analyze clinical parameters. The median follow-up time was estimated using the reverse Kaplan–Meier method. Biochemical progression-free survival and overall survival were assessed using the Kaplan–Meier method. All statistical analyses were performed using Microsoft Excel (version 16.0) and GraphPad Prism (version 10.5.0).

The Ethics Committee of the Medical Faculty of the University of Heidelberg confirmed the analysis S-193/2024. The ethics committee has no objections to the conduct of the study.

## Results

### Patient characteristics

We retrospectively identified 21 patients with LVmPC (one patient presented with an induced LVmPC) who were treated with combined PDRT and MDRT. All patients had histologically confirmed prostate adenocarcinoma and imaging-verified bone metastases. Patient characteristics were heterogeneous (Table 1); however, more than 75 % presented with at least one high-risk feature. Median follow-up was 26 months. Overall, 33 bone metastases were treated. A single bone metastasis was present in 71 % of patients, while 24 % had two to four bone metastases at treatment initiation. Bone metastases were located in the pelvis (n = 21), lumbar spine (n = 5), thoracic spine (n = 2), and ribs (n = 4).

**Table 1:** Abbreviations: IQR = interquartile range; PSA = prostate specific antigen; ADT = androgen deprivation therapy; GnRH = gonadotropin-releasing hormone; ARPI = androgen receptor pathway inhibitor.

**Patient characteristics**	
	n (%) [IQR]
Total no. Patients	21 (100)
Age at diagnosis	
Median (IQR)	71 [5.1–13.9]
Gleason score	
6	0 (0)
7a	4 (19)
7b	4 (19)
8	5 (23.8)
9	6 (28.6)
10	2 (9.5)
PSA	
Median initial [ng/ml]	17.5 [10.3–52]
Median at RT start [ng/ml]	4 [1.2–11.4]
cT-stage	
T1	7 (33.3)
T2	4 (19)
T3	6 (28.6)
T4	4 (19)
cN-stage	
N0	16 (76.2)
N1	5 (23.8)
cM-stage	
M1b	21 (100)
Number of targeted osseous metastases	
Single	15 (71.4)
2–4	5 (23.8)
>4	1 (4.8)
systemic therapy	
ADT	19 (90.4)
GnRH-analogon	16 (76.2)
Bicalutamid	3 (14.3)
ARPI	10 (47.6)
Docetaxel	2 (upfront)

### Imaging modalities

90 % of all patients received a PSMA-PET/CT before RT (n = 19/21). The mean maximum standardized uptake value (SUVmax) of the bone metastases was 9.8 (5.1–13.4). All treated bone metastases demonstrated a corresponding morphological correlate in CT imaging. In cases without a definitive CT correlate, additional MR imaging was performed. Metastases without morphological correlate in CT or MR imaging were not treated by MDRT.

### Radiation treatment

All patients received external beam radiation therapy (EBRT). PDRT and MDRT were administered concurrently in all but one patient, in whom MDRT was initiated immediately after completion of PDRT. PDRT was delivered using standard normofractionated or hypofractionated schedules with a total 2 Gy per fraction equivalent dose (EQD2) exceeding 76  Gy. If RT of lymph node metastases was performed, it was combined with elective nodal irradiation (ENI) in a simultaneous boost concept. In analogy with institutional standards for localized PC patients, ENI was recommended to patients presenting N1 disease or a Roach score >20 % in N0 disease. [28].

All patients received image-guided radiotherapy (IGRT) and intensity-modulated radiotherapy (IMRT). In 90 % of the patients, PDRT was delivered with a total dose of 76.5 Gy to the planning target volume (PTV), with 2.25 Gy per fraction. One patient only received 31 fractions at 2.25 Gy per fraction due to a history of prior pelvic irradiation for rectal carcinoma, and one patient received 60 Gy in 20 fractions (3 Gy per fraction). Five patients with N1 or M1a disease received pelvic lymphatic RT with a total dose of 51 Gy in 1.5 Gy fractions, including a simultaneous integrated boost to 61.2 Gy to the lymph node metastases. Among patients with N0 disease, nearly half (44 %) received pelvic ENI with 51 Gy in 1.5 Gy fractions analog to the inhouse PLATIN 1 trial. [29].

A total of 33 osseous metastases were treated with RT. Of these, 15 received normofractionated treatment with a prescribed dose of 61,2 Gy to 68 Gy to PTV in 34 fractions as simultaneous integrate boost concept within the PDRT. A separate stereotactic body radiotherapy (SBRT) was used for 18 metastases, delivering 27 Gy prescribed to the 80 % isodose in 9 Gy per fraction. One bone metastasis was treated with 32.5 Gy in 5 fractions, prescribed to the 70 % isodose. The choice of treatment approach was at the discretion of the treating physician, depending on anatomical situation and target size.

### Systemic therapy

The systemic treatment of the treated cohort is highly inhomogeneous due to changes in treatment recommendations in the last decade and individual patient’s comorbidity as well as patients and providers choice. It was administered at the discretion of the referring external urologists/oncologists. More than 85 % of the patients received systemic therapy prior to start of RT. Nearly half of these patients received combination therapy (ADT + ARPI or ADT + Docetaxel) upfront. Two patients received Docetaxel upfront. 38 % were treated with androgen deprivation monotherapy, and three patients did not receive any systemic therapy upfront. The mean duration of systemic therapy before the start of radiotherapy was 3.1 months [1.8–5]. Antihormonal therapy was administered in 90 % of patients after completion of RT, evenly split between ADT monotherapy and combination therapy with ADT plus an ARPI, such as apalutamide or enzalutamide. Two patients did not receive any systemic therapy- one due to prior orchiectomy and one due to patient refusal.

### Clinical Outcome

**Fig. 1:**
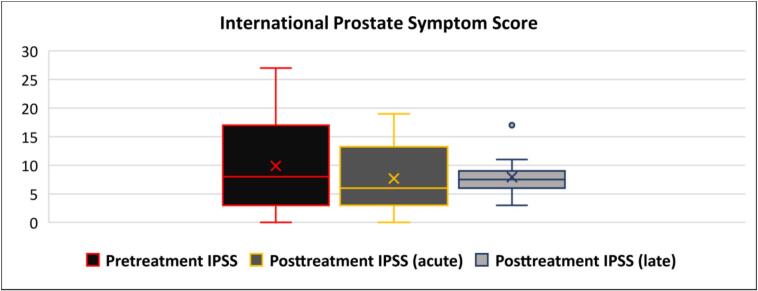
Box-plot comparing pretreatment and posttreatment IPSS.

Clinical outcomes were assessed using the CTCAE v.5.0, IPSS and the EPIC-26 questionnaires. Median and the interquartile range (IQR) of the pretreatment IPSS values (IPSS 8.5 [IQR 3–17]) were comparable to those observed during the acute (IPSS 6 [IQR 3–13]) and late post-treatment (IPSS 8 [IQR 6–9]) phases, indicating minimal overall change in urinary symptom burden over time (Fig. 1). Patients were excluded from analysis following biochemical recurrence. On average, radiation therapy resulted in a reduction of 2 points in the mean IPSS, both in the short term and at long-term follow-up.

**Fig. 2:**
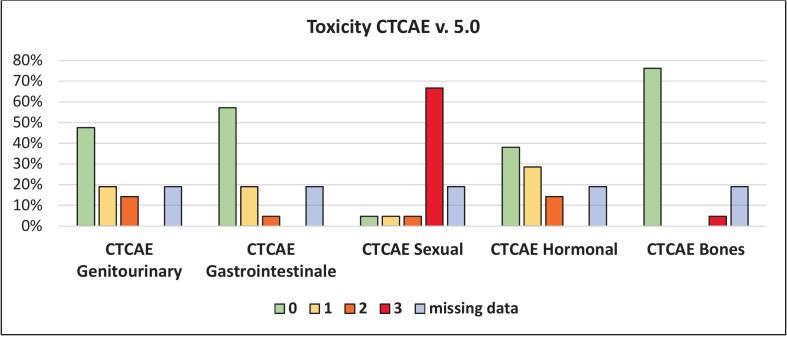
Cumulative worst toxicity from 3 months after completion of radiotherapy. Categorized into genitourinary, gastrointestinal, sexual, hormonal and bone-relating side effects.

Approximately 50 % of all patients reported no genitourinary or gastrointestinal adverse events at all more than 90 days after the end of radiation. Genitourinary or gastrointestinal toxic effects of CTCAE grade I–II were observed in 19 % of patients. No CTCAE grade III toxicities to the genitourinary and gastrointestinal tract occurred. However, 67 % of patients experienced complete loss of sexual function (CTCAE grade III). Additional hormone-related adverse effects of ≥grade II were reported in 14 % of the patients. One patient sustained an insufficiency fracture of the acetabulum (Fig. 2). Four patients were excluded of this analysis; two due to early progress and for two patients no data was available.

**Fig. 3:**
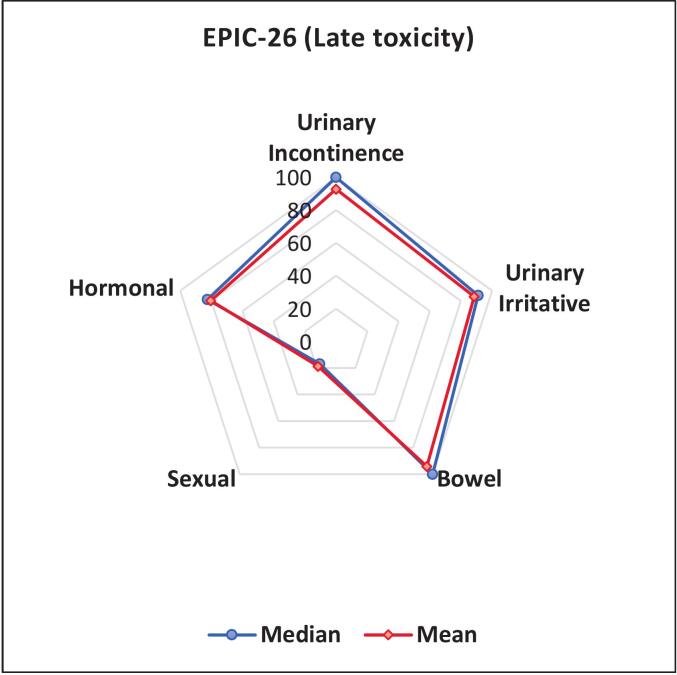
Late toxicity and impact to QoL assessed by EPIC-26 questionnaire*.*

EPIC-26 data were available exclusively in the post-RT period, owing to the retrospective study design and incomplete baseline questionnaires. Over 75 % of all patients completed the EPIC-26 questionnaire. Overall, genitourinary and gastrointestinal toxicities were mild. The mean EPIC-26-Score for urinary incontinence and irritative were 93 respectively 89. The mean bowel score was 94 (Fig. 3). Treatment-related adverse effects were most evident in the hormonal domain, whereas sexual function was largely abrogated in the majority of patients. The mean EPIC-26- score for hormonal problems was 80 and for sexual function 19.

All patients who were asked (n = 15/21) whether they would undergo RT again indicated that they would.

### Oncological outcome

**Fig. 4:**
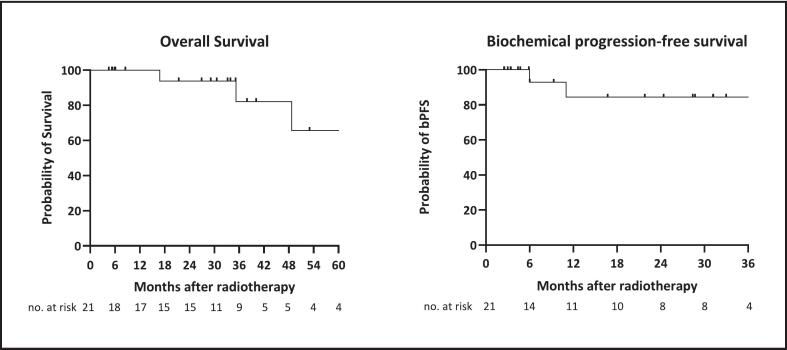
Kaplan-Meier of overall survival and biochemical progression-free survival after radiotherapy.

Following hormone ablative therapy and prior to the initiation of radiation therapy, only one patient had undetectable PSA level. Post-RT PSA values were available for more than 90 % of patients. After completion of RT, 67 % of patients reached an undetectable PSA value at any point. There was no correlation of different systemic therapies and undetectable PSA value. Undetectable PSA value was reached also by the patient that refused ADT. The time to next line systemic therapy was 7 months (11 from initiation of first systemic therapy), 13 months (19) and 40 months in the three patients who developed biochemical and radiographic progression. Notably, two of these patients had undergone only conventional bone scintigraphy for staging, whereas the remaining patients were staged using PSMA-PET/CT. Of the three recorded deaths, two were classified as disease-specific. Biochemical PFS after 36 months was over 80 % (Fig. 4). Data to evaluate RPS and LC are incomplete. For all patients for whom post treatment imaging is available, no local failure was reported. Only the patient with progressive disease seven months after end of RT already received current standard of care systemic therapy with GnRH-analogues and ARPI.

To evaluate the prognostic value of PSA nadir within the first 6 to 12 months after initiation of systemic therapy, data from 17 patients were available for analysis. Among them, 88 % (n = 15) achieved a PSA nadir ≤0.2 ng/ml without evidence of PSA progression during this interval. The two patients who did not reach a PSA nadir ≤0.2 ng/ml within 6 to 12 months were the ones who subsequently developed extensive disease progression. The third patient with reported biochemical progression had no systemic therapy before progression occurred; therefore, achieving such low PSA levels were not expected in this case.

## Discussion

Our findings suggest that combining PDRT and MDRT with systemic therapy in de novo LVmPC is feasible, well tolerated, and highly accepted by the patient.

Until the time of tumor progression, radiation-induced side effects that negatively impacted quality of life were rare. The main treatment-related adverse effects were complete loss of sexual function and other consequences of suppressed testosterone levels. Notably, no grade ≥ III gastrointestinal or genitourinary toxicities occurred, and most patients reported a stable or improved urinary symptom burden following radiotherapy, as reflected by IPSS scores.

These real-world data add important information to appropriate PSMA-PET/CT-staged patients receiving an individual RT concept radiating all PET-positive tumor sites and demonstrates the excellent tolerability with favorable risk–benefit profile.

The inconsistent results of the STAMPEDE, PEACE-1 and HORRAD trial, all adding PDRT to SOC, regarding the OS underline the necessity of further patient selection in RT in LVmPC. However, these trials demonstrate prolonged bPFS, radiographic PFS, castration resistance-free survival or reduction of serious genitourinary events. [8,9,30].

Another radiotherapeutic approach to intensifying treatment in mPC is MDRT. There are several studies showing different benefits of a MDRT in oligometastatic PC. Ost et al. demonstrated an improved PFS by MDRT compared to active surveillance. [31] Other studies could confirm the improved PFS also in addition to ADT without compromising quality of life. [15,19,32].

All of these studies suggest that RT may offer clinical benefits in the treatment of mPC. However, it remains uncertain which patient subgroups derive the greatest benefit, and whether a combination of PDRT and MDRT confers additional advantages while maintaining tolerability.

For combined PDRT and MDRT in de novo LVmPC data from prospective studies is missing. A retrospective analysis (n = 49 patients) of Imber et al. reports of favorable clinical outcomes and prolonged non-castrate remissions of a combined PDRT and MDT in addition to systemic therapy in de novo LVmPC. Robust conclusions regarding oncologic endpoints such as OS and PFS cannot be drawn due to the heterogeneity of the cohort with respect to risk factors and systemic therapies. A formal evaluation of treatment-related adverse events in this study is lacking.

The pronounced impact on sexual and hormonal function observed in our study cohort raises important considerations for the integration of radiotherapy into multimodal treatment strategies for LVmPC. While radiotherapy itself shows less high-grade sexual toxicity than the combination with prolonged ADT and, in some cases, additional ARPIs, appears to substantially contribute to long-term functional impairment. This reflects a well-known trade-off in advanced prostate cancer treatment, where oncologic control is often achieved at the expense of endocrine and sexual health.

These findings highlight the need to critically reassess the role of treatment intensity versus functional preservation, particularly in patients with limited metastatic burden who may achieve durable disease control. The concept of intermittent or time-limited ADT warrants renewed interest in combination with local therapies such as PDRT and MDRT as already demonstrated in oligoprogression. [32,33].

In our analysis, a baseline PSMA-PET/CT was performed in 19 of 21 patients which improved diagnostic certainty. As previously noted, two of the three patients with biochemical recurrence, also the two patients with prostate cancer-related death, had not undergone PSMA-PET/CT for initial staging. Although the number of events is too small to allow for statistical inference, this observation may underscore the importance of PSMA-PET/CT in accurately staging LVmPC. Given that PSMA-PET/CT imaging was not utilized in the STAMPEDE trial and is not addressed in the PEACE-1 trial, the results may underestimate the benefit of additional PDRT in LVmPC. The CHAARTED criteria used to classify low-volume and high-volume disease are based solely on conventional CT imaging. Given the increasing use of PSMA-PET imaging in daily clinical practice, it has become challenging to translate earlier data into the current diagnostic landscape. In the present study, the criteria were applied to PSMA-PET images, with the additional requirement of a corresponding morphological correlate on either CT or MRI. [11].

A comparison of the currently available data for OS, PFS or other oncological endpoints with our study is not possible because of the small, heterogenous cohort and small number of events, which restricts the ability to draw definitive conclusions. In addition, systemic therapeutic approaches were heterogeneous. One contributing factor to treatment variability was the publication of the TITAN trial, which demonstrated that the addition of apalutamide to ADT in patients with mHSPC significantly improved OS and delayed the onset of castration resistance, without compromising quality of life compared to ADT alone. Nonetheless, adverse effects related to suppressed testosterone levels—such as fatigue, skin rash, fractures, and cardiovascular events—remain a clinically relevant concern. [34].

In addition to the heterogeneous use of systemic therapy, clinical risk factors were also variable across the cohort, despite more than 60 % having a Gleason score of ≥ 8. An interesting observation was the analysis of PSA nadir within 12 months after initiation of systemic therapy, known as a prognostic marker in metastatic PC. [[25], [26], [27]] The two patients who developed extensive tumor progression did not achieve a PSA nadir ≤ 0.2 ng/ml within the first 12 months of treatment or showed ongoing biochemical progression during this period. In contrast, all other patients receiving neoadjuvant or adjuvant systemic therapy achieved PSA levels ≤ 0.2 ng/ml. Although the cohort size and number of events were limited, these findings may suggest a potential stratification marker to identify patients who could benefit from comprehensive treatment approaches, including radiotherapy to all metastatic sites.

The main limitations of this study include its retrospective design, small and heterogeneous patient cohort and variability in systemic therapy. Due to these limitations, the ability to identify predictive factors is restricted.

In summary this data demonstrates the favorable risk–benefit profile of combined PDRT and MDRT in patients with de novo LVmPC. It also sheds light on the underlying causes of QoL impairment in this setting. Prospective trials implementing PSMA-PET baseline imaging are urgently needed to identify the best integration of RT in this clinically relevant but underexplored patient population. [35].

## CRediT authorship contribution statement

**Jan-Hendrik Bolten:** Conceptualization, Methodology, Formal analysis, Data curation, Investigation, Writing – original draft, Writing – review & editing, Visualization. **Fabian Weykamp:** Investigation. **Christoph Grott:** Investigation. **David Neugebauer:** Investigation. **Lars Wessel:** Writing – review & editing. **Felix H. Englert:** Writing – review & editing. **Justus Valentini:** Writing – review & editing. **Magdalena Goertz:** Writing – review & editing. **Stephanie Zschaebitz:** Writing – review & editing. **Johannes Huber:** Resources. **Erik Winter:** Investigation. **Juergen Debus:** Project administration, Resources. **Jakob Liermann:** Project administration, Supervision, Validation, Writing – review & editing.

## Declaration of competing interest

The authors declare the following financial interests/personal relationships which may be considered as potential competing interests: This research did not receive any specific grant from funding agencies in the public, commercial, or not-for-profit sectors.

Juergen Debus received grants from Merck Serono GmbH, Accuray Incorporated, RaySearch Laboratories AB, Vision RT limited, Siemens Healthcare GmbH, Quintiles GmbH and PTW-Freiburg Dr. Pychlau GmbH outside the submitted work in the last 36 months. Jakob Liermann received travel fees from RaySearch Laboratories AB and Micropos Medical outside the submitted work.

Fabian Weykamp received speaker fees from AstraZeneca, Varian Medical Systems, Siemens Healthineers, Chulabhorn Royal Academy and Merck Sharp & Dohme and travel support for attending meetings from AstraZeneca, Varian Medical Systems, Novocure GmbH, German Center for Lung Research (DZL), Fraunhofer MEVIS, Chulabhorn Royal Academy and Micropos Medical as well as compensation for advisory boards from Novocure GmbH and Merck Sharp & Dohme.

Jakob Liermann received travel fees from RaySearch Laboratories AB and Micropos Medical outside the submitted work.

.
